# Oncolytic Adenovirus: Strategies and Insights for Vector Design and Immuno-Oncolytic Applications

**DOI:** 10.3390/v7112923

**Published:** 2015-11-24

**Authors:** Hanni Uusi-Kerttula, Sarah Hulin-Curtis, James Davies, Alan L. Parker

**Affiliations:** Institute of Cancer and Genetics, School of Medicine, Cardiff University, Heath Park, Cardiff CF14 4XN, UK; uusi-kerttulahk@cardiff.ac.uk (H.U-K.); curtiss5@cardiff.ac.uk (S.H-C.); daviesja9@cardiff.ac.uk (J.D.)

**Keywords:** adenovirus, oncolytic, virotherapy, immune epitope, neutralization, genetic masking, chimeric vector, pseudotyping, cancer immunotherapy

## Abstract

Adenoviruses (Ad) are commonly used both experimentally and clinically, including oncolytic virotherapy applications. In the clinical area, efficacy is frequently hampered by the high rates of neutralizing immunity, estimated as high as 90% in some populations that promote vector clearance and limit bioavailability for tumor targeting following systemic delivery. Active tumor targeting is also hampered by the ubiquitous nature of the Ad5 receptor, hCAR, as well as the lack of highly tumor-selective targeting ligands and suitable targeting strategies. Furthermore, significant off-target interactions between the viral vector and cellular and proteinaceous components of the bloodstream have been documented that promote uptake into non-target cells and determine dose-limiting toxicities. Novel strategies are therefore needed to overcome the obstacles that prevent efficacious Ad deployment for wider clinical applications. The use of less seroprevalent Ad serotypes, non-human serotypes, capsid pseudotyping, chemical shielding and genetic masking by heterologous peptide incorporation are all potential strategies to achieve efficient vector escape from humoral immune recognition. Conversely, selective vector arming with immunostimulatory agents can be utilized to enhance their oncolytic potential by activation of cancer-specific immune responses against the malignant tissues. This review presents recent advantages and pitfalls occurring in the field of adenoviral oncolytic therapies.

## 1. Adenovirus Immunogenicity

### 1.1. Introduction

**Figure 1:**
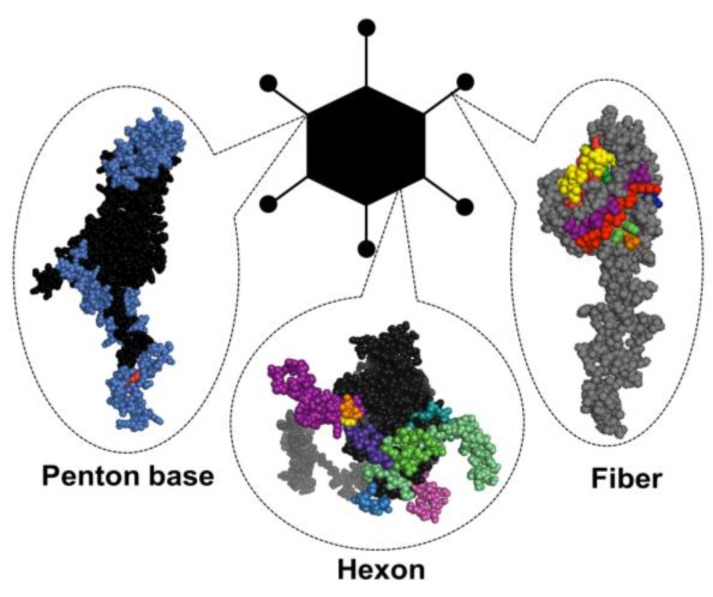
Adenovirus particle with the three major antigenic capsid proteins. Penton base, hexon and fiber (shown here as monomers) are the main building blocks of the capsid structure, but also contain the major immunogenic epitopes (highlighted in colors) that are explained in greater detail in Section 1.3 of this review.

Adenoviruses (Ads) are non-enveloped viruses containing a linear double-stranded 34–36 kb DNA genome within an icosahedral capsid. They belong to the *Adenoviridae* family. Human Ads were historically classified into species A–G based on their DNA homology, hemagglutination, oncogenic and neutralization properties, with species D containing the largest number of different Ad serotypes (for a comprehensive review, see [1]). Systematic sequence analyses have yielded detailed information on the evolutionary relationships between different Ad serotypes and enabled the classification of the 57 human Ad serotypes based on alignment of the main capsid proteins—fiber, hexon and penton base (Figure 1). Twenty species D Ads have recently been fully sequenced and found to exhibit great diversity in the hypervariable regions (HVRs) of the capsid proteins [2]. The extensive variability within the species D is suggested to be a result of natural homologous recombination, a common mechanism responsible for viral genome fitness and diversity due to selective immune pressure in the human host, which is likely to result in novel variants with altered tropism and virulence [2].

Ad viruses are constantly circulating in the human population, with small seasonal fluctuations. They are capable of infecting individuals of all ages, although babies and young children are most affected due to previous seronegativity. Ad infections commonly result in non-life-threatening conditions including mild upper and lower respiratory tract infections, gastroenteritis, cystitis or keratoconjunctivitis (Table 1) but can in rare cases produce manifestations with high morbidity and mortality such as hepatitis, pneumonia, meningoencephalitis and myocarditis (reviewed in [3]). Opportunistic infections are seen in severely immunosuppressed patients and allogeneic stem cell (or organ) transplant recipients, in whom the consequences can be fatal due to severe inflammatory host responses, cytokine “storms” and extensive immune attacks, as was demonstrated tragically in 1999 when a young volunteer patient receiving gene therapy for ornithine transcarbamylase deficiency died as a result of a cytokine storm following intravascular delivery of a huge dose (3.8 × 10^13^ viral particles) of Ad5 [4]. This case provided a significant low point in the clinical development of Ad as a biotherapy, and highlighted the requirement to better understand and refine the dose-limiting interactions that limit efficacy and promote toxicity clinically. Disseminated infections are generally associated with high liver and kidney toxicity, and may result in multi-organ failure due to high virus burden in the blood. Ad infections in immunocompetent and -compromised patients are discussed in detail in a recent review by Lion and colleagues [3].

Ad was originally isolated from a human adenoid tissue sample in 1953 [5]. The past 60 years have provided compelling evidence of the tumor-killing (oncolytic) potential of Ad-based vectors. They infect both dividing and non-dividing cells, and can incorporate large transgenes to their genome without the risk of integration into the host genome, and are therefore not considered to be intrinsically oncogenic (unlike some integrating viral vectors). Due to the generally mild nature of the disease manifestation and long clinical history, Ads are widely considered to be safe delivery vectors for gene therapy applications. The Ad genome is well-characterized, compact and largely permissive for a plethora of genetic modifications. Hence novel vector candidates are continuously being assessed for effective and safe future therapeutics. The latest trends in Ad gene therapy have included oncolytic Ads (OAds) with a variety of cancer-specificities, tumor vaccines, cancer immunotherapies and modulation of immune checkpoint control mechanisms (for reviews on oncolytic Ad-based therapies, see [6,7,8,9]).

Ad serotype 5 (Ad5) has been the most commonly used delivery vector for experimental and clinical purposes, and comprises the majority of gene therapy trials worldwide [10]. However, its wider use is severely limited by high levels of pre-existing humoral immunity in the general population, estimated to be >90% in certain geographical locations such as in sub-Saharan Africa [11,12,13]. Neutralizing antibodies (nAbs) directed against the primary antigenic epitopes in the three major capsid proteins rapidly opsonize and target the systemically-delivered therapeutic vector for elimination, thus severely hampering its therapeutic efficacy. This review aims to provide an insight into Ad-mediated immunity and implications for OAd vector design for immune evasion and selective immune modulation for successful anti-cancer therapies.

### 1.2. Tissue Tropism

Ads are capable of infecting a wide variety of vertebrate hosts via aerosol droplets in the respiratory, urinary or gastrointestinal tract (reviewed in [1]), utilizing multiple cellular entry receptors in a serotype-dependent manner (Table 1). The most widely used cell attachment receptor for all Ad species, except species B viruses, is the human coxsackie and adenovirus receptor (hCAR). It belongs to the family of immunoglobulin-like surface molecules and co-localizes in the tight junctions with zonula occludens-1 (ZO-1) protein on polarized epithelial cells (reviewed in [14]) and is ubiquitously expressed in all human organs and on erythrocytes [15,16]. Following a natural respiratory tract infection with wild type Ad5, an early event involves the over-expression of the Ad fiber protein which disrupts tight junctions, thus facilitating viral translocation from the basolateral to apical surface of the epithelial cells and enabling onward virus spread [17]. Recently, species B Ads were divided into B1 and B2 subclasses based on their differential receptor usage with species B1 utilizing the membrane cofactor protein CD46 as their primary cellular attachment receptor, whilst species B2 utilize Desmoglein-2 (DSG-2) [18]. Ad11 has been shown to utilize both CD46 and DSG-2. Ad3/7/11 binding to DSG-2 has been shown to stimulate epithelial-to-mesenchymal transition (EMT) that is a central event in carcinogenesis [18], which may limit their use as anti-cancer vectors.

**Table 1:** Human adenovirus classification, receptor usage and tissue tropism. hCAR, human coxsackie and adenovirus receptor; DSG-2, desmoglein-2; HSPG, heparan sulfate proteoglycan; VCAM-1, vascular cell adhesion molecule 1; SR, scavenger receptor; MHC1, major histocompatibility complex class 1; SA, sialic acid; nd, not determined.

Human Adenovirus Classification and Tropism			
Species	Serotypes	Receptors	Tropism
A	12, 18, 31	hCAR	Cryptic (enteric, respiratory)
B1	16, 21, 35, 50	CD46 [19], CD80, CD86	Respiratory, ocular
B2	3, 7, 14, 34, 35	DSG-2 [18], CD80, CD86	Renal, ocular, respiratory
B1/2	11	CD46, DSG-2 [18]	Ocular, respiratory
C	1, 2, 5, 6	hCAR [20], HSPG, VCAM-1, SR, MHC1-α2	Respiratory, ocular, lymphoid
D	8‒10, 13, 15, 17, 19, 20, 22‒30, 32, 33, 36‒39, 42‒49, 51, 53	SA [21], CD46 [22,23], hCAR [22], GD1a glycan [24]	Ocular, Enteric
E	4	hCAR	Ocular, respiratory
F	40, 41	hCAR	Enteric
G	52	nd	Enteric

Whilst Ads primarily utilize the fiber knob:hCAR-mediated pathway for cell entry *in vitro*, tropism *in vivo*, at least following systemic delivery (widely considered as the holy grail for oncolytic applications), appears to be dictated by a variety of other factors including the high affinity interaction between human coagulation factors (in particular Factor X, FX) that largely determines their hepatic tropism following contact with the bloodstream [25,26] (reviewed in [27]). These “adapter molecules”—multiple vitamin K-dependent coagulation factors—play a central role in Ad5 hepatotropism [28,29] that is mediated by a specific multi-factor interaction between hexon HVRs and heparan sulphate proteoglycans (HSPGs), expressed on the surface of hepatocytes. This high affinity, Ca^2+^-dependent interaction is effectively “bridged” by FX [25,30], a serine endopeptidase circulating free in the blood at a concentration of approximately 8 μg/mL. Ad5 HVR5, HVR7 (and possibly HVR3), located on the top of the hexon trimer [25,31], are known to bind to the γ-carboxylated glutamic acid (Gla) domain of the FX molecule, guiding the extensive liver sequestration of Ad5-based vectors [25].

More recently, a potentially critical role for the FX/hexon interaction has been described that protects the Ad5 capsid from complement-mediated immune attack and destruction by natural antibodies, therefore casting further doubt over the role of FX as a hepatotropic adaptor or in protection of the Ad5 virion from immune attacks [32]. Notably, species D Ads have been shown to exhibit low or abolished affinity to FX and subsequently reduced liver transduction relative to Ad5 [25], which is likely to have important implications for design of novel vector candidates based on these serotypes.

### 1.3. Structural Basis of Immunogenicity

The major antigenic epitopes of the Ad particle are located within the main capsid proteins fiber, penton and hexon. Herein, the major antigenic determinants of the well-characterized and most commonly deployed serotype, Ad5, are discussed in further detail.

#### 1.3.1. Hexon

**Figure 2:**
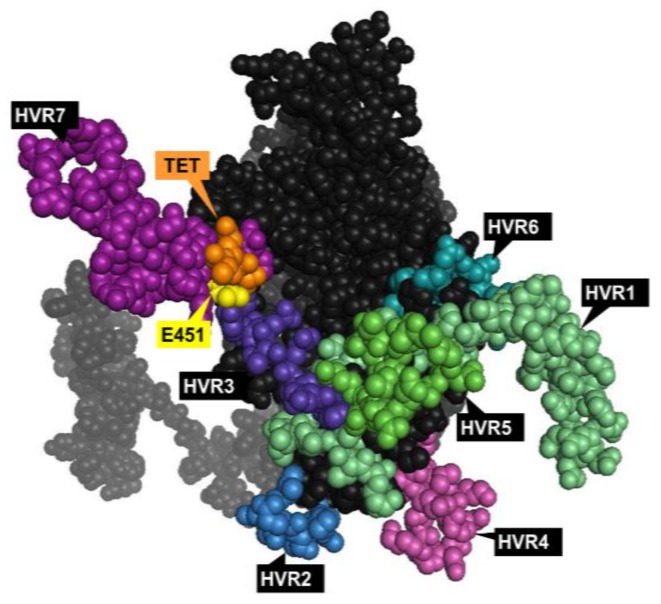
Top view of the Ad5 hexon monomer with the major antigenic epitopes. Hypervariable regions (HVRs) 1‒7 [33] shown in different colors; the site for point mutation (E451Q) central for ablation of human coagulation factor X (FX) binding [26] is shown in yellow; TET motif central for the high affinity interaction with coagulation factor VII (FVII) shown in orange [38]. The model was generated in PyMol version 1. 1eval (PDB ID: 3TG7).

The homotrimeric hexon is the largest (~130 kDa) and most abundant capsid component, with 240 pseudo-hexagonal trimers lining the 20 facets of the virion surface. Hexons can be classified into H1–H4 based on their interactions with neighboring penton proteins. They have up to nine HVRs [33] (dependent on the serotype) on the surface-exposed epitopes of the molecule (Figure 2), which represent major antigenic targets for nAbs following virus challenge [34,35]. Anti-hexon nAbs can act at both the extracellular level to sterically limit cellular association, or at the intracellular level to prevent virus uncoating and nuclear entry of viral DNA [36]. It has also been suggested that innate anti-Ad5 immune responses can be triggered by recognition of FX bound on the hexon protein [37], while a recent study reports the role of FX in shielding the Ad5 capsid from immune attacks and subsequent neutralization by natural IgM and complement proteins [32].

Additionally, Ad5 hexon HVRs have been suggested to be the specific sites determining sensitivity to the described innate immune neutralization [39], particularly following intramuscular (i.m.) vaccination or vector challenge [40,41]. The hexon harbors the main neutralizing epitope ε that can be utilized for serotyping by neutralization tests, while the γ determinant in the fiber knob is responsible for hemagglutination properties. However, resulting from extensive intra- and interspecies recombination, diagnostic typing is often non-exclusive due to cross-reactivity in hemagglutination tests [42].

#### 1.3.2. Fiber

**Figure 3:**
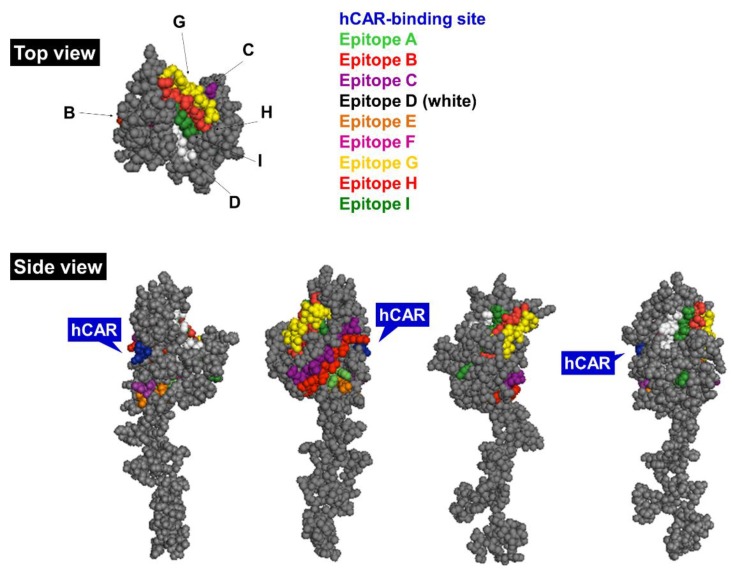
Side and top view of the Ad5 fiber monomer. Main antigenic epitopes are highlighted in distinct colors (see legend) and hCAR-binding site is indicated in blue [43]. The model was generated in PyMol version 1.1eval (PDB ID: 1KNB). hCAR, human coxsackie and adenovirus receptor.

The Ad5 fiber is a homotrimeric ~62 kDa protein located on the 12 vertices of the icosahedral capsid. It consists of a globular knob domain containing the receptor-binding domain that mediates binding to the native receptor hCAR [20] (residues Ser408, Pro409, Tyr477 and Leu485) and distinct protruding loop structures with the nine main antigenic epitopes (A‒I) involved in elicitation of serotype-specific nAbs [43] (Figure 3). The C terminus of the fiber is a rigid shaft structure, mediating contact to the underlying penton protein that plays a central role in endosome-mediated entry into host cells via αvβ3/5 integrins (entry pathway reviewed in [1]).

The fiber plays a key role in mediating viral endosome-cytoplasm transition through the detachment of the fiber protein and its membrane lytic activity in the low pH endosome. Both the length and flexibility of the fiber protein are central in viral cellular uptake, which has been demonstrated by pseudotyping the long Ad5 shaft with short shafts from other serotypes resulting in reduced infectivity and attachment [44], as well as by introducing point mutations into the KKTK motif within the fiber shaft showing impaired flexibility and drastically reduced cell transduction [44,45]. The KKTK motif was previously considered to be involved in Ad5-mediated hepatic delivery via HSPG interactions [46,47]. More recent studies, however, demonstrate this motif to be non-essential for this interaction [48], and rather that the KKTK motif exists at an essential “hinge region” within the shaft protein, where modification of this motif renders the fiber inflexible and unable to interact with cellular αvβ3/5 integrins [49,50].

The exact target of anti-fiber nAbs remains a hot topic of debate, with compelling evidence that the antigenic loops within the fiber knob form the major immunodominant domains [40,51], at least following a native Ad5 infection [52]. Ad entry into the airway epithelia results in over-expression of the fiber protein [17] and thus directly exposes the fiber to the innate immune recognition mechanisms as it is the most abundant viral component in the early stage of infection. There is significant controversy surrounding the determination of primary antigenic determinants of the Ad5 virion, as earlier reports suggest that the fiber protein does not play a central role in nAb production [53], while more recent studies have demonstrated its involvement in eliciting the majority of anti-Ad5 nAbs following a natural inoculation [52]. The role of anti-hexon and -fiber nAbs is discussed further in Section 1.6.

#### 1.3.3. Penton base

**Figure 4:**
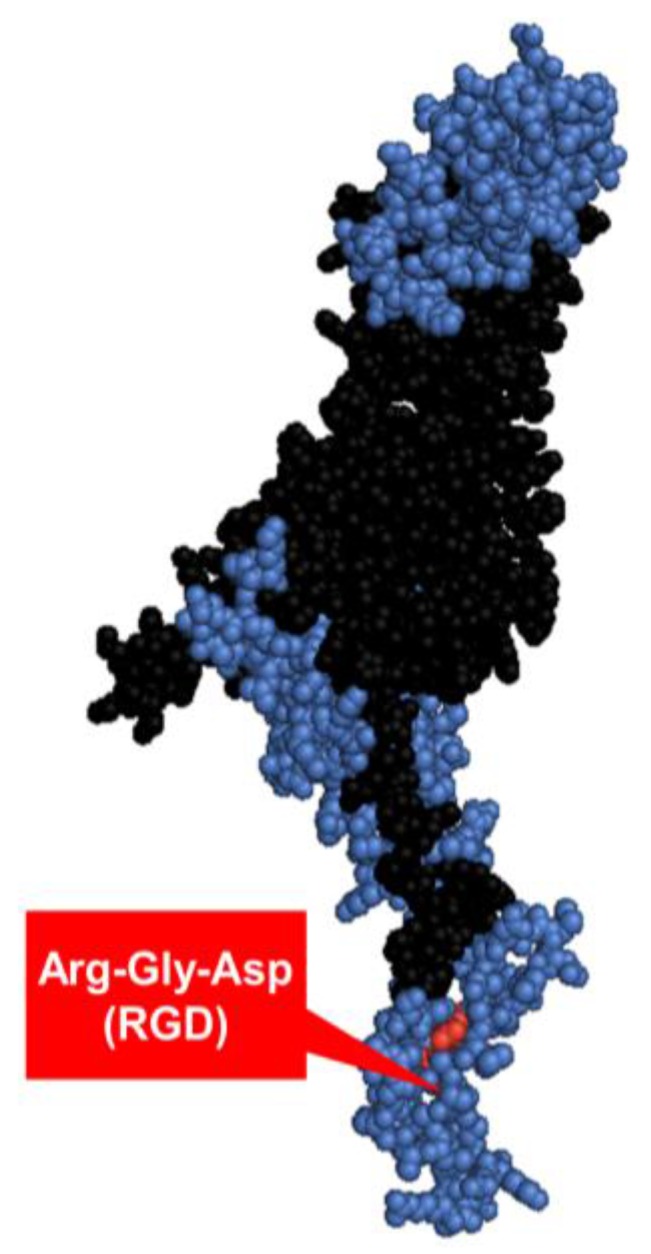
The Ad2 penton base monomer. The three main antigenic sites [57] are highlighted in blue, integrin-binding motif Arg-Gly-Asp (RGD) labelled in red [56,58]. The structure was constructed in PyMol version 1.1eval (PDB ID: 1X9P).

The third major building block of the viral capsid is the penton base, a homopentameric ~82 kDa protein (Figure 4) located on the base of each fiber trimer. While the fiber is responsible for Ad5 particle attachment on the host cell surface, the penton is involved in viral internalization, mediated by binding to the vitronectin-binding αvβ3/5 integrins on the cell surface [54] via the conserved integrin-binding Arg-Gly-Asp (RGD) epitope (amino acid residues 485‒488 [55,56]). The penton has three immunodominant neutralizing epitopes—one of which overlaps with the RGD motif—that contribute to virus neutralization mainly at the internalization step of the infectious cycle [57]. Anti-RGD antibodies have been suggested to have a non-neutralizing role in Ad infection [57].

Ad5 binding to cellular αvβ3 integrins on marginal zone macrophages (MZMΦs) via the penton RGD motif has been shown to contribute to innate immune activation by triggering the IL-1α activation and subsequent IL-1RI-dependent induction of cytokine and chemokine C-X-C motif 1 (CXCL1) and CXCL2 production, as was demonstrated in a study utilizing an Ad5 vector with RGD motif deletion (Ad5RGDΔ) [59]. Ad5ΔRGD vectors with Ad35-pseudotyped fiber (short shaft [60]) have been shown to have retained cellular attachment but an impaired internalization, which may indicate a role for this motif in endosomal escape and the importance of penton–integrin interactions for CD46-utilising species B Ads [61]. Additionally, Ad5 vectors carrying a point mutation that abolishes integrin-binding (RGD/RGE) has been shown to result in reduced splenic uptake and attenuated inflammatory responses in mice [62]. These observations suggest a central role for RGD motif not only in the viral internalization, but also in the activation of anti-Ad innate immune responses.

### 1.4. Innate Immune Responses

The three major capsid proteins, early proteins E1A, E1B, E2 and E4, and virus-associated non-coding RNAs VA-I and VA-II, have all been implicated to be central in the synergistic activation of innate immune responses that lead to inflammation and vector clearance (reviewed in [63,64]). Following systemic Ad5 vector administration, the virus is rapidly recognized and coated by proteins of the complement cascade [65,66], Kupffer cells (KCs), platelets, erythrocytes [15] and IgM antibodies that readily recognize repetitive pathogen structures such as virus capsids [67] (*in vivo* blood interactions reviewed in [27]). Human (but not murine) erythrocytes express hCAR and complement receptor 1 (CR1) and are thus capable of rapid coating and inactivation of the Ad5 particles following systemic delivery [15]. Ad5-based vectors are efficiently opsonized by complement factors—even in the absence of nAbs—which indicates central involvement of this pathway in both innate and adaptive arms of anti-Ad immunity [65]. Rapid chemokine and cytokine production, as well as Ad5-induced thrombocytopenia mediated by p-selectin and von Willebrand factor (vWF) [68], are all elicited in a C3-dependent manner, with the help of other complement factors [66] such as C2 and C1q, (reviewed in [27]). Furthermore, Ad5 is extensively sequestered by the liver [69], leading to acute transaminitis, vascular damage and even severe toxicities [70].

The spleen is another major off-target site for Ad vectors. Systemic Ad delivery results in vector binding to β3 integrins via the penton RGD motif on MZMΦs and leads to accumulation in the spleen, which initiates IL-1α-mediated activation of chemokine- and complement cascade, promoting polymorphonuclear leukocyte (PMN) activation and local inflammation [59]. Recent publications have also reported novel roles for the non-inflammatory factor of the coagulation pathway, namely FX, indicating its involvement in Ad5 virus shielding from neutralization by natural IgM antibodies and subsequent activation of the complement cascade [32]. Conversely, decoration of the virus particle with host FX has also been suggested to function as a pathogen-associated molecular pattern (PAMP), triggering innate immune responses via the Toll-like receptor / nuclear factor κB (TLR/NF-κB) pathway [37].

### 1.5. Adaptive Immune Responses

Therapeutic Ad vector delivery results in long-lasting humoral and cellular immune responses in the human host [71]. Antigen-presenting cells (APCs)—dendritic cells (DCs) and immature macrophages—recognize PAMPs and direct DC maturation and T cell activation, leading to subsequent adaptive immune responses against the pathogen (reviewed in [72]). Ad infection elicits activation of NK cells [73] cytotoxic CD8+ T cell [74] and memory CD4+ T cell responses [75]. In addition to containing major nAb epitopes as described earlier [34,35,74], the highly conserved regions of the hexon protein have been identified to contain at least three major CD4+ T cell epitopes [75,76]. Systemic production of serotype-specific IgM, IgA and IgG [77] and nAb seroconversion has been reported to occur within 2‒4 weeks of the primary virus exposure [78]. There is evidence that the intensity of humoral immune responses may be determined by vector administration site and pre-existing nAb titers rather than the therapeutic dose [79]. Different human and animal serotypes have been shown to have minimal levels of cross-reactivity, as nAbs seem to be specific to the homologous virus [80].

Anti-Ad nAbs comprise primarily fiber-, penton- and hexon-specific IgG1, IgG4 and IgA antibodies (reviewed in [72]) that can inactivate the virion at different points of its infectious cycle [34,53]. Anti-fiber nAbs seem to contribute to the blocking of initial virus attachment on the cell surface by extracellular virion aggregation [81], while anti-penton nAbs may be responsible for inhibiting the internalization step [57] and a proportion of anti-hexon nAbs potentially account for intracellular, post-entry neutralization [36], although this remains controversial. It has been demonstrated that 1.4 individual serotype-specific anti-hexon nAbs are needed for full neutralization of Ad2 [82]. Intriguingly, this study also describes how the neutralization efficiency of anti-penton nAbs can be increased from 50% to 100% efficiency by lowering the pH from 7 to 5, which is presumably due to the exposure of the penton antigenic epitopes and subsequent interaction with anti-penton nAbs.

### 1.6. Natural *vs*. Induced Immunity

Anti-fiber nAbs have been shown to emerge first after the delivery of therapeutic Ad vector, followed by generation of anti-penton nAbs and anti-hexon nAbs, suggesting that all three are needed for a full synergistic neutralizing effect [83]. However, there has been considerable controversy surrounding the specificity and immunogenicity of the capsid protein-induced nAbs. In addition to nAbs having specificities against different capsid proteins, they also seem to vary based on the route of inoculation. Reports have suggested a primary role for anti-hexon nAbs in vaccine-induced immunity [34] and anti-fiber nAbs following natural infection [52,84]. According to Cheng and colleagues, nAbs elicited by replication-deficient Ad5 vaccine vectors in seronegative individuals were directed against capsid components other than the fiber, while nAbs elicited by natural infection with wild type viruses mainly were directed against the fiber. Previously seropositive individuals had nAbs against both fiber [84] and other capsid components after vaccine challenge. On the contrary, a recent study reports only subtle differences between the specificity of nAbs relative to the infection type (vaccination *vs.* natural), suggesting the immunodominance of hexon and a subdominant assisting role for anti-fiber nAbs in neutralizing immunity [40].

The understanding of pre-existing Ad-mediated immunity is crucial for therapeutic vector design, which has been demonstrated by unsuccessful clinical trials in the past. An Ad5-based non-replicating vector MRK-Ad5, expressing HIV-1 clade B proteins Gag, Pol and Nef, was used as an HIV-1 vaccine candidate in the phase II, double-blind, randomized, placebo-controlled STEP trial involving ~3000 volunteers at 34 sites in North America, the Caribbean, South America and Australia, that was initiated in 2005 but was halted shortly afterwards [85]. Unexpectedly, the incidence of HIV-1 infection was reported 2.3-fold higher in the individuals with pre-existing anti-Ad5 immunity, relative to the respective placebo group, while the risk was not increased in seronegative individuals (reviewed in [86]). These observations were completely unexpected as the connection between the serological status and susceptibility to HIV-1 infection remains unclear. This strongly demonstrates the necessity for fully understanding the underlying factors that affect the immunogenicity of Ad vectors carrying heterologous components intended for gene therapy or vaccine applications.

### 1.7. Prevalence of Pre-Existing Humoral Immunity

Ad5 is a common respiratory virus infecting human populations worldwide. Due to its high seroprevalence, the majority of human populations carry anti-Ad5 nAbs that are highly capable of neutralizing the virus upon a secondary infection or re-exposure to a therapeutic vector. The highest seroprevalence rates of anti-Ad5 immunity are reported in the developing world, sub-Saharan Africa in particular [87], while pre-existing nAbs levels against rare human serotypes from subgroups B and D appear to be substantially lower in the studied populations [11,12,87].

**Figure 5:**
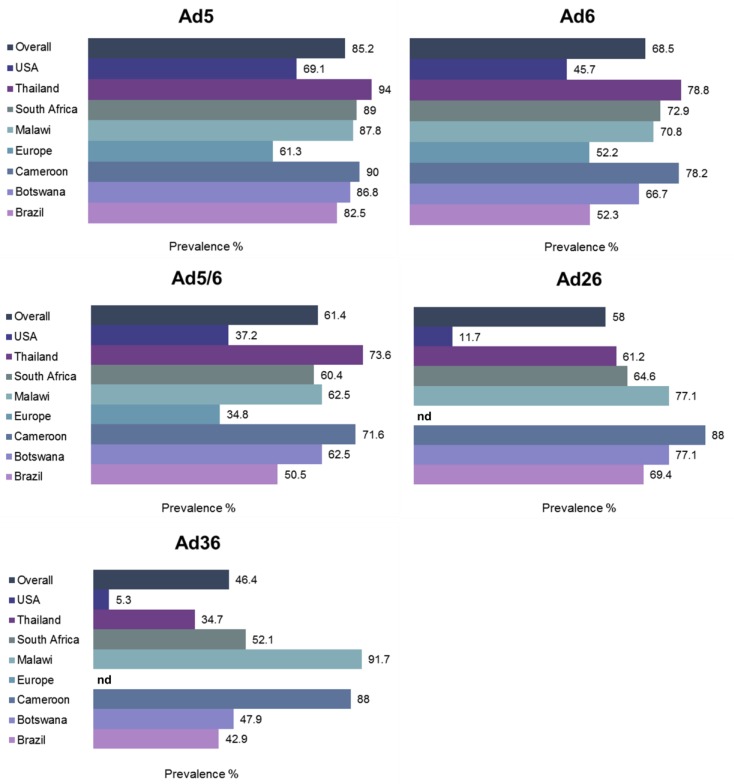
Global prevalence of neutralizing antibodies (nAbs). nAb titers against five different Ad viruses from subgroups C (Ad5, Ad6] and D (Ad26, Ad36] were determined from ~1900 individuals at 34 different locations in South America, Africa, Asia, Europe and United States. nd, not determined. Adapted from [12].

The prevalence of nAbs directed against subgroup C viruses Ad5 and Ad6, and subgroup D viruses Ad26 and Ad36 was evaluated in an international study [12] that revealed overall high prevalence of pre-existing anti-Ad5 immunity (85.2%), Thailand having close to 100% prevalence (Figure 5). Both subgroup D viruses had remarkably lower seroprevalence, overall values being 58% for Ad26 and 46.4% for Ad36. Several studies have evaluated the age-dependence of anti-Ad immunity in different populations. In pediatric populations in sub-Saharan Africa, maternally-derived anti-Ad5 Ab titers were high until six months of age, then remained low until the age of two years then increasing rapidly until seroprevalence rates reached adult levels by seven years of age [88]. It is thus crucial to take into account the variable seroprevalence rates between different Ad serogroups when selecting new Ad vector candidates for patients from different age groups, particularly when intended for systemic delivery.

### 1.8. Vector Neutralization by Ovarian Ascites

Due to the significant challenges associated with achieving targeted tumor specific delivery of Ad via the systemic route, localized intratumoral (i.t.) delivery would circumvent many of the dose-limiting vector–host interactions. Ovarian cancer is the leading cause of death from gynecological cancers and the fifth most common cancer in women in the UK [89]. The advanced stage of the disease is characterized by high frequency of metastases and build-up of malignant ovarian ascites that represents the chemo-resistant and recurrent disease with poor prognosis (reviewed in [90]). Large volumes of fluid cause major discomfort and pain in the patients, requiring routine drainage (paracentesis) to temporarily ease the patients’ symptoms [91]. Ovarian cancer presents an appealing target for OAd virotherapy due to the potential of direct vector administration intraperitoneally (i.p.), thus having access to both primary and metastatic tumors, as well as other tumor-associated cell types residing in the ascitic fluid.

Ascites has a protein content similar to serum, including substantial levels of anti-Ad5 nAbs [92] and is highly capable of neutralizing Ad5-based vectors [93,94]. In a study by Hemminki and colleagues, levels of nAbs were compared in both serum and ascites following i.p. delivery of Ad5 vector into ovarian cancer patients. Induction of nAb production was rapid in both serum and ascites following viral delivery, and nAb titers were not affected by the administered dose. Surprisingly, Ad transgene expression was relatively unaffected by ascites, albeit likely due to 4-fold lower nAb titers in ascites as compared to serum nAb titers [92]. Removal of the fluid prior to OAd virotherapy may help reduce vector neutralization and improve bioavailability for active tumor targeting, thus improving efficacy. Ascitic fluid contains diverse cell populations, one of which is epithelial ovarian cancer cells (EOC) that can be readily cultured from the patient-derived ascites [95,96]. These *ex vivo* cultures represent an important platform for functional analyses of ovarian cancer cell properties [97] and assessment of the interplay between the fluid and its components may provide essential insight into the possibilities for treating disseminated peritoneal cancers with Ad-based i.p. therapies in the future.

### 1.9. Oncolytic Adenoviruses—the Clinical History

Ads are inherently lytic against their host cells, releasing the virus progeny to the neighboring cells. OAds can be generated by a number of genetic engineering strategies by combining attenuation of genes essential for viral replication to achieve high selectivity and safety, and incorporation of genes that are expressed only in neoplastic cells for the generation of cancer-specific vectors. Insertion of cancer-selective promoters is a strategy that can be utilized to generate conditionally-replicative Ad vectors (CRAds), promoting restricted lysis of cancer cells with defective signaling pathways and/or characteristic tumor microenvironment. Ad vectors have undergone three generations of development, involving the deletion of early genes E1, E2, E3 and E4 that are central for viral replication, viral DNA transcription/replication of late genes, modulation of immune responses and metabolism of virus mRNA/host protein synthesis, respectively. The first generation Ads were modified to carry an E1/E3 mutation, a basic attenuating mutation for generation of replication-deficient vectors, while second generation Ads had an E4 deletion in addition to E1 and/or E3 deletions (reviewed in [6,98]).

The past decade has marked the approval of the first oncolytic Ad5-based vector ONYX-015 for treatment of head and neck cancers in China [99]. In healthy tissues, tumor antigen p53 is the guardian of DNA damage and stress, and activates DNA repair mechanisms, initiates apoptosis, prevents angiogenesis and controls excessive cell proliferation by cell division arrest at the G1/S stage of the cell cycle, among its other diverse functions (reviewed in [100]). Mutations in the *p53* gene can lead to uncontrolled cell division and carcinogenesis, which is why its proper functionality is crucial for the maintenance of healthy tissues. ONYX-015 vector carries a complete deletion of the *E1B* 55 kD gene (Δ1520) that renders it capable of selectively infecting neoplastic cells with a defective p53 tumor suppressor pathway [101]. However, only an estimated 50% of all cancer patients carry mutations in the *p53* gene [100]. Therefore, complementing strategies are urgently needed for cancer patients who have a functional p53 pathway and are thus refractory to these treatments. On the contrary, ~97% of ovarian cancer patients with high-grade serous (HGS) carcinoma have been tested positive for defective p53 pathways in large scale genomic analyses [102,103], highlighting the potential of OAd-based therapies targeting this pathway in ovarian cancer.

It has been suggested that the retinoblastoma protein (pRB) tumor-suppressor pathway may be defective in all human cancer types [104], which indicates the feasibility of targeting this pathway for cancer therapies [105]. A 24-base pair deletion (Δ24; dl922‒947) in the pRB-binding domain of *E1A* has been shown to efficiently restrict viral replication in proliferating cells with a defective pRB pathway [105], enabling transition from G1 to S phase [104]. An Ad5-based oncolytic ICOVIR-5 targeted to tumors with a defective pRB pathway, combines *E1A* transcriptional control by EF2 promoter mutation, Δ24 mutation and an RGD-4C modification in the fiber HI loop [106,107], is currently undergoing phase I clinical studies for treatment of advanced melanoma and has shown promising safety and anti-tumor efficacy [107]. An oncolytic HYPR-Ad5 vector developed by Post and colleagues utilizes hypoxia-dependent E1A expression and subsequent selective lysis of hypoxic cells that are the dominant population of cells resistant to chemo- and radiotherapies in solid tumors [108].

Recently, a novel Ad5 vector ORCA-010 carrying a novel mutation (T1) has shown enhanced oncolytic efficacy and safety in pre-clinical *in vivo* studies, and is estimated to be significantly more potent than the licensed ONYX-015 vector [109]. The T1 mutation contains insertion of a single adenine base at position 445 within the endoplasmic reticulum (ER) retention domain of the *E3/19K* gene that has been shown to greatly enhance oncolytic potency of Ad5, presumably due to increased release of virus progeny from the ER and efficient spread within the tumor mass [110]. ONCOS-102—a chimaeric Ad5/knob3 vector armed with the potent immunostimulatory granulocyte-macrophage colony stimulating factor (GM-CSF)—has completed phase I clinical studies with increased cancer-specific CD8+ T cell responses and good safety profile in heavily pre-treated patients with injectable solid advanced tumors [111] and is ready to enter phase II/III studies. Phase I studies demonstrated enhanced immunologic response, good safety and a significant correlation between anti-viral and anti-tumor T cells, while neither safety nor efficacy were affected by the Ad5/3 fiber pseudotyping [112].

Rapid elimination of the therapeutic vector from the bloodstream following systemic delivery occurs frequently via recognition by pre-existing anti-Ad5 nAbs, which severely limits the use of this serotype for oncolytic applications and thus the use of alternative serotypes is required. Recently, a novel “directed evolution” approach was used to create the first wholly non-Ad5-based oncolytic species B Ad. ColoAd1 (also known as enadenotucirev, EnAd), a complex and highly potent chimaeric Ad3/Ad11p virus (Δ*E1A/E1B*), was generated through forced evolution via recombination of a pool of Ads from different subgroups on tumor cell lines. ColoAd1 has a small 24 bp deletion in the region encoding E4orf4 that, in the context of Ad5, induces protein phosphatase 2A (PP2A)-mediated, p53-independent apoptosis [113]. However, the oncolytic mechanism of ColoAd1 seems to be entirely apoptosis-independent and its enhanced potency may be related to the chimeric E4 region that may alter the expression of additional, yet unknown genes [114]. ColoAd1 has shown significantly improved selectivity and cancer-killing as compared to the ONYX-015 [114], and phase II/III clinical trials are currently ongoing for the treatment of colon cancer and other solid tumors. Furthermore, superior blood persistence and oncolytic activity was observed for ColoAd1 in the highly neutralizing environment of the whole human blood, which indicates its potential suitability for i.v. treatment of disseminated tumors [115].

Ad vectors are highly potent immunotherapeutic agents and can successfully be utilized for prime-boost regimens. However, their efficacy as monotherapies remains frustratingly low and therefore the combination of potent Ad vectors with existing gene-, chemo- and radiotherapies is likely to result in better therapeutic responses due to multiple mechanisms of cell killing, eradication of the malignant tumor tissues and long-lasting systemic responses (reviewed in [116,117]). Despite of the recent advances in the Ad virotherapy field, major challenges still remain. It is clear that innovative strategies for vector design, coupled with selective immune stimulation within the tumor microenvironment, hold significant promise for effective cancer eradication.

## 2. Therapeutic Vector Design for Host Immune Evasion

### 2.1. Introduction

Ad vectors elicit extensive anti-viral humoral and cellular immune responses in their host, which severely impair the clinical outcome of oncolytic virotherapies due to premature clearance of the therapeutic vector. Successful Ad vector development should therefore involve careful consideration of viral particle masking strategies in order to achieve reduced immune recognition and improved bioavailability for the target tissue. The Ad vector can be modified genetically or chemically, to carry adapter molecules, or pseudotyped to contain small domains or whole capsid proteins from less seroprevalent human and animal serotypes, in order to combat the hostile neutralizing environment encountered in the human body. One of the novel approaches in the oncolytic virotherapy field is “biological” shielding of reovirus vectors with potent immune cells [118]. Intriguingly, the decorated reovirus particles were not only protected from pre-existing nAbs present in ovarian ascites, but were also capable of combining efficient oncolysis with immune priming by inducing innate and specific anti-tumor adaptive immune responses. This study describes a novel dual cancer therapy that could potentially be adapted to Ad-based applications.

### 2.2. Ads with Low Seroprevalence

Anti-Ad5 humoral immunity may be circumvented by using less common serotypes that have low natural seroprevalence in the general human population. Additionally, utilization of other than species C Ads is appealing due to their variable receptor and tissue tropisms, as the expression of the primary species C receptor hCAR has been shown to be down-regulated in progressive cancers [119,120], and may therefore not be the optimal tumor target. Ad vectors have been extensively modified by exchanging specific capsid components with those from less seroprevalent or immunogenic serotypes (virus chimeras reviewed in [121]). Chimeric or pseudotyped vectors with altered cell selectivity, antigenicity and tissue tropism have been successfully tested in both pre-clinical [11,35,52,53,122,123] and clinical trials [107,111,114] over the past decades.

Ad subgroup D has the largest diversity of viruses, coupled with other advantages such as low seroprevalence rates [11] and decreased intrinsic hepatotropism due to low affinity to FX [25]. They appear capable of infecting their host cells via multiple surface receptors, such as CD46 [22,23], hCAR [22], sialic acids [21] and GD1a glycan [24], whilst the involvement of additional receptors has not yet been ruled out. A recent study compared 16 different species D Ads for their ability to spread in tumor mass [124]. Ad9 was found to be the most efficient at infecting both hCAR-negative and -positive cells, and thus presents an appealing alternative serotype for cancer applications. Barouch and colleagues demonstrated Ad35, -26 and -48 vaccine vectors to be more immunogenic in rhesus monkeys than the hCAR-utilizing Ad5, resulting in high levels of innate anti-viral and pro-inflammatory cytokine responses [125]. In a recent study by Camacho and colleagues, species D viruses Ad26, -28 and -48 were shown to be more efficient at cell transduction via intranasal delivery, while Ad5 was the most efficient in i.m. delivery [126]. Importantly, i.m. transduction levels were restored in mice ubiquitously expressing human CD46. Additionally, Ad26 had significantly improved DC transduction as compared to Ad5 that has been shown to only infect these cells via a lactoferrin and DC-SIGN-mediated pathway [127].

An interesting study compared the replication efficacy and oncolytic potency of 15 different species B, C, D and F viruses in B cell cancer cell lines and primary B cell cancers [128]. Species D viruses were shown to have the overall most efficient oncolysis in a panel of B cell cancer cell lines, primary patient marginal zone lymphoma cells, and primary patient CD138+ myeloma cells *in vitro*. Ad26, -45 and -48 showed markedly improved oncolysis compared to all other viruses (including Ad5), while a single i.t. administration of Ad26 and Ad45 resulted in a significantly reduced growth of *in vivo* lymphoma xenografts.

Mastrangeli and colleagues compared intratracheal administration of Ad4 (species E) and Ad30 (species D) with Ad5 administration, demonstrating no cross-reactivity between the viruses from different subgroups [129]. In this study, Ad4 and Ad30 were capable of efficiently transducing the airway, regardless of a prior exposure to Ad5. According to the authors, these findings may have potential translational applications in the treatment of respiratory manifestations of cystic fibrosis to overcome the pre-existing immunity in the airways, when repeated administration is required. The same group approached the problem by using an Ad2 vector from subgroup C, showing that pre-existing immunity was indeed efficiently circumvented but the persistence of transgene expression was compromised [130].

#### 2.2.1. Pseudotyped/Chimeric Vectors

Ad vectors have been modified by switching the major capsid components into domains from less seroprevalent or immunogenic serotypes (for a review, see [121]). Substituting Ad5 hexon and fiber domains containing the major immunogenic epitopes with those from rare serotypes, is becoming a common vector design strategy and multiple candidates are assessed in a number of pre-clinical studies [35,52,53,122]. Exchange of Ad5 hexon HVRs with low seroprevalence Ad48 HVRs (Ad5HVR48] has been shown to result in altered vector immunogenicity in recent studies. An Ad5HVR48 vector expressing simian immunodeficiency virus Gag protein efficiently escaped anti-Ad5 nAb responses and showed comparative immunogenicity to the parental Ad5 [35]. However, subsequent *in vivo* studies by Coughlan and colleagues with Ad5HVR48 showed significantly increased toxicity and immunogenicity in mice following i.v. delivery, despite favorably decreased hepatotropism [131]. More recently, Teigler and colleagues demonstrated that Ad5HVR48 was indeed capable of evasion from pre-existing anti-Ad5 nAbs but displayed reduced hepatotoxicity and had similar T cell responses to Ad5, suggesting improved safety and reduced immunogenicity for this vector, relative to Ad5 [132]. These conflicting observations highlight the unpredictable nature of chimeric Ad vectors and reinforce the importance of rigorous preclinical evaluation to fully delineate the immunogenic properties of the recombinant vectors.

Another widely exploited genetic modification approach is the swapping of either the Ad5 fiber knob domain or complete fiber protein with rare Ad serotypes. Fiber pseudotyping has been shown to result in altered innate [133] and adaptive immune activation [30,134], which implicates an important role for this genetic modification strategy in Ad vector design. However, a study performed 20 years ago reports that Ad5 fiber replacement with Ad7 (species B) fiber did not change the immunogenicity of the vector, despite successfully altered tropism [53]. Similarly, Ophorst and colleagues have reported increased DC transduction and T cell activation *in vivo* using an Ad35 fiber-substituted Ad5 vector, but no protection from anti-Ad5 immunity [122]. In contrast, Särkioja and colleagues achieved substantial protection from nAbs in mice treated with fiber-modified Ad5 vectors [52]. In this study, Ad5 fiber knob domain pseudotyped with Ad3 fiber knob (Ad5/3) showed increased gene delivery in presence of low and high nAb titers, in comparison to Ad5. Additionally, while anti-Ad5/3 nAbs were able to neutralize Ad5 only marginally, anti-Ad5 nAbs neutralized Ad5 and blocked gene delivery entirely. However, higher nAb titers induced by triple immunization with Ad5/3 were capable of cross-neutralization of Ad5, which indicates the involvement of other neutralizing epitopes either within fiber shaft or other capsomers.

Denby and colleagues pseudotyped Ad5 vectors with fibers from a species B virus Ad16 and two species D viruses Ad19p and Ad37 and showed equal or improved transduction of vascular endothelial and smooth muscle cells (SMCs), as compared to Ad5 [135]. Parker and colleagues evaluated a panel of pseudotyped Ad5 vectors with fiber derived from subgroup D Ads Ad17, -24, -30, -33, -45 and -47 and showed that these vectors directly interacted with FX and efficiently transduce the liver, thus demonstrating that the interaction with FX is not fiber-mediated [48]. However, neither of the above-mentioned studies compared the antigenicity of the fiber-pseudotyped vectors, which would be an intriguing next step in assessment of these candidates for therapeutic applications. An interesting study by the same group reports that pre-existing immunity can be partially bypassed by pseudotyping Ad5 with fiber from species D virus Ad45 (Ad5/F45) [30] that had previously shown high levels of FX-mediated cell binding and transduction [48]. This study demonstrated greatly improved protection for Ad5/F45 from nAbs in the presence of 2.5% sera that largely neutralized the control Ad5 vector, indicating that nAbs are at least partially directed against the fiber protein and may thus contribute to extracellular neutralization [82].

As for pseudotyping with subgroup B viruses, an Ad5-based Ad35 fiber-chimeric virus (Ad5T*F35++) was shown to exhibit improved vascular cell transduction and protection from anti-Ad5 nAbs in human sera [136]. In this study, 33% of 102 human serum samples neutralized Ad5 by a minimum of 90%, while only 18% of the sera were capable of neutralizing Ad5T*F35++ at similar levels. In addition to having the fiber protein from Ad35, Ad5T*F35++ carries a mutation (T*) that ablates FX interactions [137], for combining potential immune evasion with efficient liver de-targeting, which is an important consideration in Ad vector design. Furthermore, when the Ad35 fiber pseudotyping was coupled with penton pseudotyping (Ad5/F35/P35), this vector was capable of transducing human SMC cultures *in vitro*, intact mouse aortas from CD46-transgenic mice *ex vivo* as well as human saphenous vein *ex vivo* at significantly higher levels than either of the control vectors Ad5 or Ad/F35 [138]. The promising results suggest potential utility of this strategy for vascular gene therapy and other tissues with high levels of CD46 but low levels of hCAR expression.

Accelerated, random evolution may be utilized for generation of a pool of potent recombinant viruses with altered virulence, tropism and immunogenic properties. This novel strategy mimicking the inherent random recombination occurring between different Ad serotypes, has recently been introduced into the field of Ad vector design (discussed in Section 1.9). Multiple Ad serotypes from various subgroups were passaged on a panel of cancer cell lines, subjecting them to random recombination and thus directing the emergence of a chimaeric Ad3/11p serotype (ColoAd1) [114]. ColoAd1 has shown superior oncolytic properties on colon cancer cell lines as compared to the previously developed OAds [114], and has paved its way for phase I/II clinical trials. The vector carries a large deletion in the E3 region, a small 24 bp deletion in the E4 region and a chimaeric Ad3/Ad11p E2B region such that the pTP and DNA pol regions have been swapped to those from a species B1 virus Ad3. Its major capsid proteins appear to originate from a species B2 virus Ad11p, which suggests uptake via the CD46/DSG-2 pathway. ColoAd1 has been found to have a superior blood persistence, as its oncolytic efficacy was only marginally deteriorated in pooled human sera and whole blood [115]. Evidently, that is a highly advantageous feature for an oncolytic vector intended for i.v. treatment of disseminated solid tumors.

Taken together, the synergistic role of anti-hexon and anti-fiber humoral immunity indicates that HVR substitutions are not likely to fully overcome the constrains of pre-existing immunity [84]. The penton protein plays a pivotal role in the early steps of Ad infection through αvβ3/5 integrin binding, and is involved in the early stages of anti-viral immune recognition [54,59], as discussed in Section 1.3.3 of this review. Modifying the penton RGD motif may therefore have important implications in the modulation of Ad vector design. αv integrin-binding mutation RGD/RGE has been shown to lead to 5-fold reduced uptake in splenic uptake and drastically reduced antiviral inflammatory responses [62]. Conclusively, it may be beneficial to combine penton ΔRGD or RGD/RGE modifications with hexon and fiber substitutions and tropism-modifying mutations for development of vectors capable of efficient immune escape.

#### 2.2.2. Non-Human Vectors

Non-human or “xeno” Ads have been evaluated for potential use as therapeutic vectors and are gaining increasing interest due to three advantages over human Ads: (1) lack of pre-existing neutralizing immunity; (2) relatively efficient transduction of human cells and (3) altered tissue tropisms. The variability in receptor usage is a major advantage that could allow targeting of various clinically-relevant cell types and thus bypassing the limitations of hCAR-mediated cell entry. Additionally, animal Ads have very low pathogenicity in their host, are replication-defective in human cells and can tolerate longer exogenous DNA inserts (transgenes) than human Ads. The use of non-human Ad serotypes such as canine Ad2 (CAV-2), bovine Ad3, porcine Ad3, ovine Ad7, murine Ad1, several simian, and fowl Ads as potential gene delivery vehicles is discussed in great detail in a recent comprehensive review [139]. Notably, Kremer’s group have successfully studied CAV-2 for the treatment of neurological diseases due to its low immunogenicity, selective CAR-mediated neuronal transduction, efficient axonal spread and long-lasting bioavailability for the mammalian brain (reviewed in [140]). Due to these advantageous features, this vector may hold potential for the treatment of tumors located within the central nervous system (CNS) and thus warrants further investigation.

Several studies have reported strategies for non-human Ad or pseudotyped chimeric vector engineering. Bradley and colleagues showed that an Ad5-based vector with HVRs substituted from Ad48 serotype and fiber substituted from a chimpanzee virus AdC68 (utilizes CAR) largely evaded pre-existing anti-Ad5 nAbs in neutralization assays with mouse and human sera while retaining its functionality [40]. Ovine [141] and porcine Ads [142] have been shown to infect human and murine cells at comparable levels to human Ads, and to be protected from neutralization by pre-existing anti-human Ad nAbs. The lack of cross-reactivity has potential implications for vector re-administration for prime-boost vaccine regimens, as alternating the delivery of human Ad- and supplementing animal Ad-based vectors could be used to overcome vector neutralization that frequently renders viral gene therapy inefficient [143]. In addition to neutralization evasion, their CAR-independent tropisms may open new avenues for the treatment of various target tissues. A study describes a chimeric Ad5 pseudotyped with canine Ad2 fiber that was shown to transduce CAR-deficient cells at 30-fold increased efficiency relative to Ad5, but fails to report any potential alterations on vector immunogenicity [144].

### 2.3. Genetic Masking

Tropism-modification within the main capsid proteins, achieved by genetic engineering to incorporate short heterologous targeting peptide sequences, has been widely investigated (reviewed in [27,145]). These strategies could potentially be utilized for masking of the Ad antigenic epitopes alike, as the insertion of peptides could favorably shift antigenic recognition away from the major antigenic epitopes. The fiber protein has been the most frequently modified for these purposes [52,146], due to the location of major antigenic epitopes within the knob domain [43], its native role in primary cell tethering, and its tolerance of genetic manipulation.

#### 2.3.1. Heterologous Peptide Incorporation within the Fiber

Previous exposure to Ad elicits the production of anti-Ad nAbs that rapidly recognize and eliminate systemically-administered Ad vectors from the bloodstream. However, pre-existing immunity should also be taken into account when localized delivery into distal cancer sites or metastases is anticipated. In the context of ovarian cancer applications, vector neutralization by nAbs present in ascitic fluid may be circumvented by genetic or chemical masking of the viral capsid, the fiber in particular. Blackwell and colleagues reported successful evasion of nAbs in ascites using a tropism-modified Ad5 vector with an integrin-binding RGD motif insertion in the fiber knob [94]. Our research has utilized a similar masking approach by genetically inserting short high affinity peptides targeted to receptors over-expressed on various tumors into the Ad5 fiber knob HI loop [146]. In this study, an Ad5 vector with a 12-mer epidermal growth factor receptor (EGFR)-targeting peptide insertion demonstrated up to 700-fold improved transduction in patient-derived primary EOC cells (hCAR^high^/EGFR^high^) and protection from pre-existing nAbs in the presence of 2.5% highly neutralizing ascites as compared to the parent vector Ad5.Luc. Future vector design strategies will focus on combination of pseudotyping with targeting peptide insertions within the main capsomers, with the view of improved immune evasion and simultaneously enhanced tumor-targeting.

#### 2.3.2. Fiber Deknobbing

Another genetic fiber-modification strategy is “deknobbing”, the removal or replacement of the fiber knob domain with artificial peptide structures. This technique has been utilized in the context of tropism-modification and vector re-targeting into integrin-expressing cells [147]. The Ad5 vectors carried a deleted knob domain and the last 15 shaft repeats, and had a genetically inserted external trimerization motif coupled with an integrin-targeting RGD motif. The authors report impaired infectivity, observed as delayed viral spread *in vitro*, but successful vector transduction into hCAR-deficient cells, as well as retained functional integrity of the fiber protein. Intriguingly, while an anti-knob antibody was capable of hampering the infectivity of the wild type Ad5, the deknobbed virus retained its infectivity in the presence of the antibody. The study warrants further investigation and detailed assessment of its antigenicity and immunogenicity, and describes a potential vector platform for further modification for gene therapy purposes.

Belousova and colleagues designed complex fiber chimeras combining an affibody targeting human epidermal growth factor receptor type 2 (Her2), a major tumor marker [148]. The Her2-specific affibody has a three-helix bundle domain Z derived from *Staphylococcus* protein A, thus replacing the trimeric knob structure, fused to the carboxy-terminal fragment of the T4 phage fibritin protein. The foldon domain of the fibritin (Fc11) is connected to the affibody with a flexible peptide linker, thus enabling correct trimerization and receptor binding of this artificial fiber protein. The authors report that the proteins were expressed at equal levels to Ad5, and fully functional virions were successfully targeted to Her2-expressing cells, thus suggesting the feasibility of this strategy for cancer-targeting. Another strategy has employed replacement of the Ad5 fiber knob domain with a small chain T cell receptor (scTCR) that is specific to the melanoma-associated cancer-testis antigen MAGE-A1 presented by HLA-A1, coupled with an extrinsic trimerization motif (Ad5.R1-scTCR) [149]. This OAd vector not only showed ablated native tropism, but was also capable of efficient killing of MAGE-A1(+)/HLA-A1(+) melanoma cells in an epitope-specific manner, thus demonstrating rigorous cell-specificity and potential applicability for treatment of melanoma.

### 2.4. Chemical Shielding

OAds have proved successful in selective killing of cancer cells whilst sparing normal cells by being engineered to “hijack” the cancer cell`s altered tumor-suppressive cellular machinery. Releasing progeny virions that spread across adjacent tissue to amplify the Ad effect, local Ad administration has proved successful in clinical trials [150]. H101 Ad has been approved for use in China to treat head and neck cancer (for a review, see [151]), while systemic delivery of OAds for solid and metastatic tumors has proved more of a challenge. A major limitation in a clinical setting is the interaction of Ad with components of the blood including macrophages, DCs, platelets, erythrocytes and KCs. In particular, interaction with FX results in reduced bio-distribution through re-directed Ad vector tropism to the liver. The resulting hepatic toxicity limits the use of high doses of OAd [152,153,154]. As described in detail earlier, activation of the host innate immune system in response to Ad capsid proteins facilitates Ad clearance coupled with activation of humoral immunity by the presence of pre-existing nAbs, significantly reducing vector efficacy. Another important limitation is the dependence of Ad for cell entry via the native hCAR [15,20]. hCAR expression is frequently down-regulated in many cancers [120,155], in particular advanced ovarian cancer [156,157,158], currently limiting the use of OAd in this setting.

Technological advances to balance effective targeting of Ads to cancer cells whilst avoiding reduced Ad bio-availability through immune activation *in vivo* are now emerging. A number of strategies are being developed (for a review, see [7]). Nanotechnology represents a plethora of gene delivery vehicles where the potential for drug (or Ad) delivery via nanoparticle encapsulation or conjugation to nanoparticle carriers is possible (for an excellent review, see [159]). Chemical shielding using a number of distinct carriers to bypass the negative effects of Ad delivery *in vivo* has been extensively studied. Some investigators have utilized multiple strategies to optimize gene delivery and minimize immune surveillance. Chemical shielding by covalent linkage via amine chemistry on the Ad capsid, aim at reducing hepatocyte tropism through evasion of binding with blood components, in particular FX and nAbs, and extending serum half-life.

#### 2.4.1. PEGylated Polymeric Carriers

First described by O`Riordan and colleagues [160], chemical modification of Ad by covalent attachment of polyethylene glycol (PEG) to hexon and fiber, has been shown to reduce immunogenicity, prevent nAb binding, increase solubility and Ad serum half-life. Chillón and colleagues shielded the negatively-charged Ad particles with cationic GL-67/dioleoylphosphatidylethanolamine-PEG (GL-67/DOPE-PEG) and achieved improved cell transduction and protection from nAbs *in vitro* [161]. Disappointingly, the vector was not protected from immune attacks *in vivo* as it was efficiently neutralized when delivered into immunized mice. Another study reports the conjugation of Ad5 with metoxypolyethylene glycol succinimidyl propionate (MPEG-SPA), which resulted in 10-fold improved nAbs evasion but dramatically decreased transgene expression as compared to the non-PEGylated virus, likely due to compromised hCAR-binding [162]. The same authors successfully generated a 5-kDa PEGylated Ad5 that showed reduced levels of nAb production with increased anti-tumor efficacy against metastatic lung cancer in mice [163].

PEGylation of Ad can prevent FX interactions, thus reducing hepatotoxicity and facilitating escape from phagocytic cells [164]. A negatively-charged, non-immunogenic molecule, PEG is hydrophilic and thereby increases solubility *in vivo* (for a review, see [165]). However, the addition of sufficient PEG to mask FX binding sites on the capsid can substantially reduce cellular transduction efficiency due to the ensuing bulkiness of modified Ad and reduced access of Ad ligands for target cell attachment. In an attempt to overcome this, Suzuki-Kouyama and colleagues engineered PEGylated Ad vectors using an avidin-biotin interaction within the hexon [166]. This proved unsuccessful as aggregation of Ad vectors due to the strong affinity of avidin for biotin reduced Ad cell transduction. Subsequently the same group targeted the Ad hexon for PEGylation but exploited the high affinity of FX interaction as a hexon-specific adaptor molecule [164]. PEG conjugation to lysine residues on FX led to random PEGylation and resulted in replacement of PEG-FX with endogenous FX *in vivo*. They suggest future studies should focus on creating site-specific PEGylation of FX.

Prill and colleagues demonstrated a chemical shielding approach that can be modified to either target or de-target hepatocytes according to the size of the PEG moiety [167]. They generated a small number of PEG polymers (750 Da in contrast to 10 kDa molecules) specifically directed to the HVR5 of the Ad hexon that significantly reduced hepatocyte transduction after i.v. delivery. However, it is hypothesized that this approach may fail in those individuals that have nAbs against regions of the capsid other than the HVRs [165]. Highlighting the complexities of targeted gene therapy, Doronin and colleagues demonstrated that a systemically administered OAd showed reduced hepatocyte transduction when conjugated with 20 kDa PEG in comparison to a 5 kDa PEGylated OAd when hepatocyte transduction was observed after 24 h [168]. The authors propose that Ad hepatocyte transduction can occur via the integrin pathway via the RGD penton base (hCAR-independent)—an effect that was inhibited by the larger 20 kDa PEG. The level of tumor cell transduction was similar in 20 kDa PEGylated Ad and un-PEGylated vector and both demonstrated efficacy by tumor elimination.

#### 2.4.2. Bio-Reducible (Cationic) Polymers

Polymer-based strategies, including the use of bio-reducible polymers, have been studied in depth in the non-viral field (for a review, see [169]), with a number of applications adapted for the Ad field. In the context of cancer gene therapy applications, coating the Ad5 particle with an arginine-grafted bio-reducible polymer (ABP) significantly increased Ad cellular transduction in both hCAR^low^ and hCAR^high^ cells *in vitro* [170]. The cationic ABP-coated Ad complex exhibited a significantly reduced immune response as measured by macrophage-derived IL-6 release in comparison to the (naked) control Ad, suggesting the use of hybrid vectors comprising both viral and non-viral DNA as a promising gene therapy approach. The authors subsequently hypothesized that the size of their Ad-ABP complex might be too large for efficient cellular uptake and may cause off-target sequestration [171]. They subsequently produced a cationic polymer (mPEG-PEI-g-Arg-S-S-Arg-g-PEI-mPEG (PPSA) containing both bio-reducible disulphide bonds and functional arginine moieties that reduced cytotoxicity and enhanced cancer cell transduction both *in vitro* and *in vivo*. More recently, coating of two OAds with 5-amine polymers conjugated with 3.4K PEG (OAd/M3.4kPN5LG) or 5K PEG (OAd/M5kPN5LG) showed the strongest killing effects in cancer cells in comparison to OAds coated with biopolymer alone or naked Ad [172].

In an attempt to reduce potential immunogenicity due to residual linker groups left at the amine attachment site after release of the polymer, Prill and colleagues constructed a cysteine residue at HVR5 (AdHexCys) that allowed attachment of thiol-based groups (allowing reversible and irreversible interactions) in addition to a synthetic shielding poly-[*N*-(2-hydroxypropyl)-methacrylamide] (pHPMA) polymer [173]. Fisher and colleagues constructed an Ad5 vector coated with the multivalent hydrophilic pHPMA, coupled with re-targeting ligands for basic fibroblast growth factor receptor (FGFR) and vascular endothelial growth factor receptor (VEFG) [174]. This vector not only exhibited improved protection from nAbs, but also efficiently infected receptor-positive cells *in vitro* and xenografts *in vivo*. The authors describe this method could be used for conjugation of a wide range of targeting molecules including peptides [175] and biological effectors, in order to generate vectors with altered tropism and immunogenicity.

Expanding the principles of multiple strategies for enhanced OAd delivery to cancer cells, Kim and colleagues produced an OAd expressing short hairpin RNA against IL-8 (Ad-∆B7-U6shIL8) to (measurably) reduce the immune response against this vector [176]. Using a new biodegradable poly cystaminebisacrylamide-diaminohexane [poly (CBA-DAH)] (CD), low cytotoxicity and high efficiency of cellular transduction was achieved. The construct was conjugated with a RGD motif in the penton base to exploit αvβ3/5-mediated cell internalization and further conjugated to PEG500. The authors demonstrate an enhanced integrin-dependent apoptotic effect in a cancer cell line with the Ad/CD-PEG500-RGD, an effect independent of hCAR. Furthermore, this vector showed suppression of IL-8 and VEGF expression.

#### 2.4.3. Liposomes

Liposomes have been studied extensively for various therapeutic purposes, and have been adopted in some studies related to OAd administration. Delivery of Ad using an emulsion containing a cationic lipid 1,2-dioleoyl-3-trimethylammonium propane (DOTAP) enhanced delivery of Ad to mouse and human cancer cell lines in comparison to DOTAP liposomes. However, Ads were most efficiently transduced in an emulsion containing an oil Lipiodol with 5 KDa PEG [177], an example of another gene delivery vehicle. This is termed lipid-polymer hybrid nanoparticles (LPNs) (for a review, see [159]). Combining a polymer core encapsulating the Ad with a surrounding lipid layer enveloping the polymer core (for biocompatibility) and an outer PEG (chemical shield) provides an excellent gene delivery vehicle. Of particular note is the controlled release capabilities from the polymer core, a function of potential interest for some gene therapy applications.

#### 2.4.4. Mechanical Means of Ad Delivery into Tumors

Ad vector delivery may be enhanced by the innovative combination of mechanical particle coating and focused ultrasound [178,179]. In a recent study, Mo and colleagues investigated the properties of passive accumulation of Ad vector into tumor tissue generated by ultrasound *in vivo* [180]. Using multi-PEGylated gold as a nanoparticle for Ad delivery, they report that the increase in density of Ad associated with the addition of gold particles enhanced focused inertial cavitation by ultrasound in comparison to Ad alone or Ad-pHPMA. This approach also maintained shielding properties. They produced a nanoparticle comprising an outer coating of 2-kDa PEG attached to an Ad conjugated with 5-kDa PEG and gold. A disulphide bond between the Ad and PEG-gold enabled dissolution of the complex in the reducing conditions of the cancer cell and infection reactivation. Using a combination of gold labelling and focused ultrasound, the authors were able to demonstrate increased tumor accumulation of viral particles from 0.1% (no gold, no ultrasound) to 12% (gold labelled, focused ultrasound) of the injected dose in the tumor following i.v. administration in mice [180]. This has a number of potential clinical advantages, not to mention the ability to potentially control the level and timing of Ad delivery—a strategy that could potentially be readily applied to OAds.

#### 2.4.5. Bi-Specific Adapter Molecules

As an alternative approach to develop Ads with altered tropism, the incorporation of bi-specific non-covalently linked adapter molecules to target Ads towards target-specific cellular receptors is under investigation. Bi-specific adapter molecules comprise an anti-Ad-fiber antibody conjugated to a target cell-specific peptide. Early studies conjugating an anti-fiber moieties to folate [181], FGF [182], EGF [183,184,185] and endothelial receptors [186] have demonstrated the potential for this targeted strategy to improve Ad transduction (for a more extensive review, see [145]).

The targeting moiety of bi-specific adapter molecules that are not integrated into the viral genome are consequently lost during viral replication. In the context of OAds, this is a significant limitation as targeting and oncolytic potential is reduced. To overcome this, van Beusechem and colleagues constructed a CRAd that incorporated an expression cassette for a bi-specific adapter molecule within the CRAd genome [184]. The bi-specific single-chain (scFv) antibody 425-S11 is composed of the anti-EGFR (scFv 425) and anti-Ad fiber knob (scFv S11). The antibody directs Ad tropism towards EGFR-positive cancer cells. After CRAd replication and oncolysis, newly released virus and antibody bind and spread to neighboring cells in an EGFR-targeted (hCAR-negative) manner. Carette and colleagues utilized the same CRAd but incorporated hCAR and integrin-binding mutations to abolish native receptor binding in an attempt to implement a stricter targeting approach (CRAd Ad∆24P*F*-425S11) [187]. The results demonstrated a significant increase in oncolysis in hCAR-deficient, EGFR-positive cancer cells.

As an alternative to the antibody-based approach, more recent studies have developed targeted strategies involving designed ankyrin repeat proteins (DARPins) that bind to the Ad5 fiber knob (mutated to ablate hCAR-binding) and fused this protein with a DARPin specific for the tumor marker Her2 [188,189]. This approach can be applied to a number of targeting strategies. The adapter contains two fused molecules, both of which contain DARPins; one binding the Ad5 fiber knob and the other with the capacity to bind to a range of cancer cell markers. Harvey and colleagues have constructed two bi-specific peptides by fusing the extracellular domain of hCAR with an EGFR- or urokinase-type plasminogen activator receptor (uPAR)-targeting polypeptide in order to achieve retargeting of an Ad5-based vector (Ad-CMV-*lacZ*) into these major tumor markers [190]. Co-administration of an Ad-CMV-*lacZ* vector with the fusion peptides resulted in significantly improved targeting into EGFR^high^/hCAR^low^ or uPAR^high^/hCAR^low^ovarian and bladder cancer tissues, relative to control Ad5. Another bi-specific retargeting strategy has exploited the high affinity interaction between the Ad5 hexon and the Gla domain of FX [25] by designing adapter molecules consisting of the Gla domain fused to ScFvs with variable specificities [185]. In this study, an Ad5 vector were successfully bridged into major tumor markers such as Her2, EGFR and the stem cell marker ATP-binding cassette protein G2 (ABCG2) via the adapter molecules, suggesting the potential for this strategy for bi-specific targeting purposes.

## 3. Vector Design for Immuno-Oncolytic Therapies

### 3.1. Introduction

The persistent dilemma in Ad cancer immunotherapies remains—how to evade immune recognition but to selectively stimulate cancer-specific immune responses. Cancer immunotherapy is a current hot topic both in immunology and cancer research, and Ad-based vectors have great promise within this area. The novel viral vector-based cancer immunotherapies commonly utilize two main mechanisms for host immune activation: (A) immune priming or cancer vaccination—lysis of infected tumor cells leading to innate immune responses and activation of adaptive immune responses against the tumor-associated antigens (TAAs); and (B) localized release of immunoregulatory agents that modulate signaling pathways defective in different cancer types (reviewed in [191]). TAAs are derived from proteins synthesized by the tumor cell, and may be either membrane-bound, secreted, cytoplasmic or localized in the nucleus [192]. Examples of commonly studied TAAs for cancer gene therapy include CD19, CD20, CD30, CD40 CD33, CD52, Her2, EGFR, VEGF, carcinoembryonic antigen (CEA), epithelial cell adhesion molecule (EpCAM) (reviewed in [193]) and human MUC-1 (hMUC-1) [194]. Immuno-oncolytic Ad vectors can potentially be used in combination with conventional chemo- and radiotherapies in order to target both primary and metastatic tumors, and to achieve an enhanced and long-lasting therapeutic effect. Additionally, they can be readily modified to carry “cargo” molecules—to express monoclonal antibodies (mAbs) directed towards TAAs, T cell receptors, immune checkpoint inhibitors (such as anti-CTL4 and anti-PD1), cytokines, and programmed to target DCs and tumor-infiltrating lymphocytes (TILs).

### 3.2. Cancer Vaccines

Ads are capable of infecting specific cell types in a receptor-dependent manner, and elicit subtle innate and adaptive immune responses, which makes them feasible cancer vaccine vectors for priming and stimulation of the host’s immune system (reviewed in [195]). Ads provide a number of potential advantages as cancer vaccines over conventional therapies. They are tumor-selective, *in situ* cancer vaccines, providing higher cancer-specificity and better safety margin. Additionally, they are able to kill cancer cells through a range of mechanisms from direct virus-mediated cytotoxicity, cell death due to anti-angiogenesis and vasculature targeting by Ads, to cytotoxic immune effector-induced cytotoxicity. This induces cell death by apoptosis, necrosis, and autophagy [196]. With the exception of apoptosis, all other types of cell death have been considered to be inflammatory and immunogenic. However, recent studies by investigators working on chemo- and radiotherapy have led to new concepts, that apoptotic cell death can be divided into “immunogenic cell death” (ICD) and “non-immunogenic cell death” [197,198]. Based on this new classification, apoptotic cell death caused by some OAds are ICD. Together, immunogenic apoptosis, necrosis and autophagic cell death caused by OAds provides a natural repertoire of TAAs in conjunction with danger signals damage-associated molecular pattern (DAMP) and Ad-derived PAMPs, as well as inflammatory cytokines [197]. Ideally, the strong elicited immune memory will then be able to recognize and attack the tumor cells in the event of a relapse or development of metastatic tumors at any anatomical location later in the patient’s life.

A number of viral vectors currently in clinical development have shown great promise for amplification of the vaccinative effect of virus infection due to the insertion of the immunostimulatory cytokine GM-CSF transgene into the viral genome [198,199,200,201]. Ad-based vectors have been engineered to express TAAs such as Her2, tyrosinase-related protein 2 (TRP-2) and gp100 [202,203] that are over-expressed in the tumor during infection, thus increasing the opportunity for immune responses to be generated towards these tumor-specific antigens [204]. However, results suggest that over-expression of a TAA is insufficient to overcome immunosuppression in the tumor or immunodominant responses against viral antigens [205]. Therefore, additional approaches are required to boost TAA-specific responses. Priming the host with a heterologous vector expressing the TAA prior to oncolytic vaccination significantly enhances the anti-tumoral response [205].

Previous studies demonstrate the potential of Ad5 to infect DCs via bridging interactions with lactoferrin, produced locally at sites of inflammation, to DC-SIGN receptors on DCs in the absence of hCAR expression [127]. This may limit the breath of immune stimulation as these cells are key actors in immunoregulation and antigen presentation. To overcome this issue, a recent study describes the generation of an Ad5-based vector with fiber knob pseudotyped with porcine Ad type 4 [206]. This chimaeric vector was shown to have improved transduction in DCs, improved tumor-specific antigen presentation and T cell-mediated IFN-γ release in mice. Xie and colleagues describe the use of a replication-deficient Ad expressing livin protein that is expressed on the surface of various cancer cell types [207]. The vector exhibited improved DC transduction that led to cytotoxic T cell activation against a panel of cancer cell lines *in vitro*.

Ad vectors have also been used as backbones for vaccines against HIV. The disappointing results from the unsuccessful STEP trial have highlighted the potential limitations of Ad5 for vaccine purposes, including for cancer applications, and therefore studies based on other serotypes appear warranted [123]. In this study, Ad26, -35, and -48 vectors expressing lymphocytic choriomeningitis virus (LCMV) glycoprotein, elicited high magnitude memory T cells, in addition to circumventing high baseline Ad5-specific nAbs. While Ad5 vectors were able to elicit similarly high memory T cell levels, they exhibited functional exhaustion and decreased anamnestic potential following secondary antigen challenge. Therefore, the use of other serotypes than Ad5 warrants further investigation, and may lead into the development of efficient and safe vector candidates in the future.

### 3.3. Immune Checkpoint Blockade

Immune checkpoint therapy, that targets regulatory pathways in T cells to enhance anti-tumor immune responses, has led to important clinical advances and provided a new weapon in the war against cancer [208]. The function of these pathways is to down-regulate T cell signaling in order to prevent uncontrolled T cell proliferation, protecting tissues from auto-immune damage and maintain tolerance to self-antigens. It is now clear that tumors co-opt certain immune-checkpoint pathways as a major mechanism of immune resistance, particularly against T cells that are specific for tumor antigens. [209]. Many of the immune checkpoints are controlled by ligand-receptor interactions, which can be readily blocked by antibodies or modulated by recombinant forms of ligands or receptors making them appealing therapeutic targets [210].

Antibodies targeting the immune inhibitory co-receptors cytotoxic T lymphocyte antigen 4 (CTLA-4) and programmed death-1 (PD-L1) have demonstrated clinical activity in a variety of tumor types, including melanoma, RCC, and NSCLC [211,212] (reviewed in [213]). As novel anticancer agents, they have a distinct profile of anti-tumor activity and toxicity, underscoring their unique mechanism of activity. Whereas CTLA-4 and PD-1 both function as negative regulators, each plays a non-redundant role in modulating immune responses [214]. CTLA-4 was the first immune checkpoint receptor to be clinically targeted, and plays a pivotal role in attenuating the early activation of naïve and memory T cells. Normally, after T cell activation, CTLA-4 is upregulated on the plasma membrane where it functions to down-regulate T cell function by outcompeting the activating receptor CD28, for its ligands, B7-1 (CD80) and B7-2 (CD86) [215]. In contrast, PD-1 is primarily involved in modulating T cell activity in peripheral tissues via its interaction with its ligands PD-L1 and PD-L2. Unlike CTLA-4, PD-1 can be found on other activated lymphocytes including B cells and NK cells. PD-L1 and PD-L2 are commonly upregulated on the surface of many different human tumors. High expression levels of PD-L1 have been shown on melanoma, lung, ovarian, and other human cancers [210,216].

The combination of Ads with a blockade of immune checkpoints is an exciting strategy that may overcome current shortcomings associated with either approach alone. The Ad and the immune-checkpoint blocker could be administered as two separate therapeutics but delivery of the checkpoint inhibitor directly from an Ad is a more appealing method. This would localize the inhibitor to within the tumor microenvironment, conferring several advantages for both safety and potency, and overcoming many of the adverse events observed with i.v. delivery. Given the importance of the immune checkpoints in maintaining immune homeostasis there is concern that a blockade of these receptors and/or ligands could lead to a break in immune self-tolerance, resulting in autoimmune/autoinflammatory side effects. In the phase III trial of ipilimumab, Grade 3 or Grade 4 immune-related adverse events (including rash, colitis, hepatitis, and endocrinopathies) occurred in 10%–15% of patients treated with the anti-CTLA-4 antibody as compared to 3% of those treated with gp100 alone. During this trial, there were 14 deaths related to ipilimumab (2.1%), 7 of which were due to immune-related adverse events [217]. Delivering the immune-checkpoint from the oncolytic virus would localize the treatment and may therefore mitigate the risks inherent in systemic delivery.

In preclinical studies of a replication-competent Ad expressing a full length CTLA-4 antibody a 43-fold higher antibody concentration in the tumor as compared to the plasma was noted. The plasma levels in treated mice remained below the reported human safety threshold [218]. Therefore, viral delivery of anti-CTLA4 mAb led to increased tumor concentrations without increase in systemic levels. In a separate study measles virus vectors encoding antibodies against CTLA-4 and PD-L1 showed therapeutic benefits in terms of delayed tumor progression and prolonged median overall survival in animal studies [219], highlighting the important, synergistic potential that combining immune checkpoint inhibition with an oncolytic viral activity may have within the future clinical cancer arena.

## 4. Perspectives

Promising data arising from the OPTiM phase III clinical trial of T-VEC (previously known as OncoVEX-GMCSF), demonstrate a durable and significantly increased response rate in patients with malignant melanoma compared to controls [199,201]. This has helped to re-energize the field of oncolytics, providing proof of concept in the technology, and demonstrating the clearest evidence to date that virotherapies are finally coming of age in the clinical arena.

Within the new era of clinical virotherapies, technologies based on Ad are likely to be heavily represented due to their ease of scale up, amenable genomes, long clinical history and potential to achieve tumor-selectivity. In order to generate the most efficacious Ad-based delivery systems, it is clear that a wide variety of considerations must be addressed. Firstly, capsid proteins must be re-engineered and optimized to evade pre-existing immunity and other dose-limiting interactions that promote uptake into a wide variety of non-target sites. Secondly, to ensure selectivity of transduction, technologies must be adopted and developed to present tumor-selective targeting ligands into the viral capsid, whilst “safety nets” must also be integrated into the viral genome to ensure replication/transgene expression is unique to cancerous cells, for example, through the inclusion of tumor-specific promoter elements or miRNA silencing elements within the viral genome. Finally, the delivery of a suitable viral payload must be incorporated into the genomic armory, whether a gene that directly or indirectly induces cell suicide in the tumor cells, or one that helps to stimulate a suitable host anti-tumor immune response.

It is therefore clear that as our knowledge of Ad systems increases, so does the degree of genetic re-engineering necessary to achieve efficient targeting increase. Therefore, the development of successful Ad-based systems for cancer applications will necessitate a highly integrated approach, and the use of high throughput systems for wholescale viral genome engineering, such as those based on recombineering, to enable progression to clinics in a timelier manner than previously considered possible.
